# Effect of Bariatric Surgery on Migraine Headache: A Systematic Review and Meta-Analysis

**DOI:** 10.7759/cureus.97294

**Published:** 2025-11-19

**Authors:** Mostafa Mahran, Oday Al-Asadi, Rofida Sobh, Dima Salloum, Almoutuz Aljaafreh

**Affiliations:** 1 Upper Gastrointestinal Surgery, Homerton University Hospital, London, GBR; 2 General Surgery, Homerton University Hospital, London, GBR; 3 Family Medicine, Ain Shams University, Cairo, EGY; 4 Obstetrics and Gynecology, American University of Beirut Medical Center, Beirut, LBN; 5 Upper Gastrointestinal Surgery, King's College Hospital, London, GBR

**Keywords:** bariatric surgery, biliopancreatic diversion, duodenal switch, gastric band, gastric bypass, migraine, one anastomosis gastric bypass, roux-en-y gastric bypass, sleeve gastrectomy, weight loss

## Abstract

Background: Migraine is a common, disabling neurological disorder, and obesity has been associated with an increased frequency of migraine, chronicity, and severity. Bariatric surgery is the most effective treatment for substantial weight loss in morbidly obese patients. We conducted a systematic review and meta-analysis (per PRISMA 2020 guidelines; registered in PROSPERO (CRD420251175413)) to assess whether bariatric procedures influence migraine frequency and severity in patients with obesity.

Method: We searched PubMed, Scopus, and Embase for studies on obese patients with a history of migraine who underwent bariatric surgery (including sleeve gastrectomy, Roux-en-Y gastric bypass, or gastric banding) and reported pre- and postoperative migraine outcomes. Six studies (n=1899 patients) met the inclusion criteria. Data on migraine frequency, intensity, and disability before and after the surgery were extracted. Outcomes were pooled using a random-effects meta-analysis, and study quality was assessed using the Newcastle-Ottawa scale.

Results: Across the six included studies (n=1899), a variety of bariatric procedures were performed (sleeve gastrectomy, Roux-en-Y bypass, and gastric banding). The pooled results showed a consistent and significant reduction in migraine frequency following surgery. All pooled effect estimates favored the postoperative state, indicating fewer monthly headache days after weight loss surgery. Individual studies have also reported a reduction in migraine pain severity and disability after surgery. The magnitude of headache improvement was correlated with weight loss. Mechanistic insights suggested that postoperative weight loss led to hormonal changes (e.g., lowered calcitonin gene-related peptide levels) and reductions in pro-inflammatory cytokines (such as IL-6 and TNF-α), all of which may contribute to the observed migraine relief.

Conclusion: This systematic review indicates that bariatric surgery substantially reduces migraine frequency and severity in obese patients. Migraine improvement likely arises from the multifactorial effects of massive weight loss, including neuroendocrine modulation and decreased systemic inflammation, rather than bariatric surgery per se. Given the high comorbidity between obesity and migraine, weight-loss surgery should be considered as a therapeutic option to alleviate the migraine burden in obese individuals.

## Introduction and background

Migraine headaches are recognized as chronic and progressive neurological disorders that may be associated with nausea, vomiting, sensitivity to light and sound, persistent head pain, and functional impairment [1]. While not commonly recognized as a contributing factor for migraines, several studies have established a connection between obesity and increased frequency and severity of migraine headaches [2]. Furthermore, obesity is correlated with the conversion of episodic migraines into chronic migraines and serves as a significant risk factor for the advancement of migraine severity [3]. In addition to resulting in adverse health outcomes and disability, migraine has a notable economic effect on the USA. This is evident through the direct expenses associated with migraine, including heightened healthcare costs, as well as indirect expenses like reduced productivity and disability [4].

The prevalence of severe obesity has significantly increased over the past ten years, and its association with migraines has been previously acknowledged [5,6]. Inflammatory substances like calcitonin gene-related peptide and cytokines, which are elevated in individuals with obesity, have been identified as mediators of pain in neurovascular inflammation, ultimately leading to the onset of migraine pain [7]. In addition, weight loss achieved via bariatric surgery has also been observed to enhance the occurrence of migraine-related symptoms and shorten the duration of headaches during episodes [2].

Bariatric surgery is presently regarded as the most successful strategy for achieving lasting weight loss when conventional methods have been ineffective [8]. The aim of the present study was to perform a systematic review and meta-analysis of the existing literature to ascertain the impact of bariatric surgery on migraine headaches.

## Review

Methods

The Preferred Reporting Items for Systematic Review and Meta-Analyses (PRISMA) 2020 guidelines [9] and the Cochrane criteria [10] were followed in the conduct and reporting of this systematic review. This review was prospectively registered in the International Prospective Register of Systematic Reviews (PROSPERO) under the registration number CRD420251175413 on October 15, 2025. The full protocol is publicly available at https://www.crd.york.ac.uk/prospero/display_record.php?ID=CRD420251175413. The protocol prespecified the objectives, eligibility criteria, and outcomes in accordance with PRISMA 2020 and Cochrane guidelines.

Study Eligibility

The present study conducted a screening process based on predefined inclusion and exclusion criteria. Studies involving patients who had undergone bariatric surgery of any type, including sleeve gastrectomy, gastric bypass, or gastric banding, with a medical history of migraine headaches and pre- and postoperative migraine assessments, were included. Any retrospective, prospective, or randomized controlled studies, case series, conference paper, or abstract. We excluded studies that had insufficient or duplicated data, a missing control arm, or a sample size of less than 10 patients.

Search Strategy and Data Extraction

A comprehensive literature search from database inception to December 10, 2025, for studies examining the effect of bariatric surgery on migraines was performed following PICO(s) criteria (Table 1).

**Table 1:** PICOS framework for the study PICOS: population, intervention, comparator, outcomes, study design Framework outlining the key components of the research question and inclusion criteria for this systematic review and meta-analysis

Component	Description
Population (P)	Patients with migraine
Intervention (I)	Patients who underwent bariatric surgery
Comparator (C)	Patients before bariatric surgery
Outcomes (O)	Frequency of migraine
Study design (S/T)	Randomized controlled trials, cohort studies, or case series (≥10 patients)

The present investigation was conducted with the utmost fidelity to a previously established methodology that had been collectively assented to by all contributing authors of the research, in conjunction with adherence to the directives outlined in the PRISMA guidelines. A systematic literature search was conducted across PubMed, Scopus, and Embase databases to ensure a comprehensive and unbiased identification of relevant studies. The following terms were used in multiple different combination (“bariatric surgery” OR “weight loss surgery” OR “roux en y gastric bypass” OR “sleeve gastrectomy” OR “Gastric bypass” OR “ one anastomosis gastric bypass” OR “Duodenal switch” OR “vertical banded gastroplasty” OR “biliopancreatic diversion”) AND (migraine) were manually searched. Two authors independently extracted data according to the predefined search strategy. Disagreements were resolved by consensus among the reviewing authors (MM, OA, DS). Outcome extracted included the frequency of migraine.

Statistical Analysis

For categorical outcomes, the odds ratio (OR) and corresponding 95% confidence interval (CI) were calculated using the random-effects model based on the Mantel-Haenszel method. Continuous outcomes were analyzed using the weighted mean difference (WMD) and 95% CI, applying the random-effects model with the inverse variance method. The random-effects model was chosen to account for potential heterogeneity among studies. All statistical analyses were performed using the Review Manager (RevMan) software, version 5.3 (The Cochrane Collaboration, London, England, UK).

Quality and Publication Bias Evaluation

The Newcastle-Ottawa Quality Assessment Scale (NOS)7 was used as an evaluation tool to assess nonrandomized controlled trials (non-RCTs) [11]. The range of scale varies from 0 to 9 stars. Studies evaluated with a score equal to or higher than 5 were considered to have adequate methodological quality and were included. There were no RCTs in the literature to be included. Two investigators (MM and OA) rated the included studies independently, and a final decision was reached by consensus comprehensive screening of the titles and abstracts of the search with utmost independence. The data obtained from each study were also extracted independently by the authors, adhering strictly to the same set of criteria.

Results

**Figure 1:**
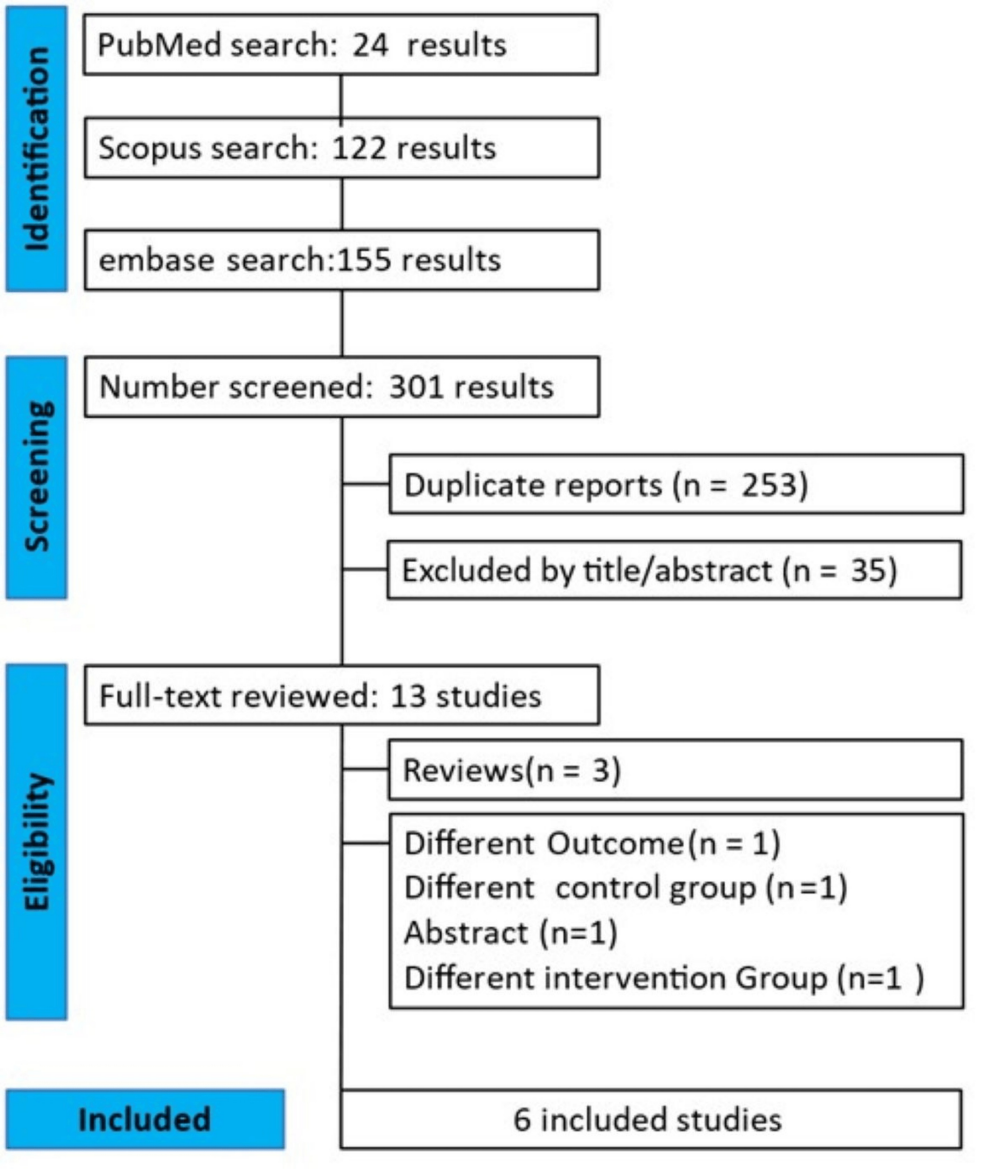
PRISMA flow diagram of study screening and selection PRISMA: Preferred Reporting Items for Systematic Review and Meta-Analyses

This is a systematic review following the PRISMA guidelines (Figure 1).

In total, 301 records were identified via database searches (PubMed: 24; Embase: 155; Scopus: 122). After removing 253 duplicates, 48 unique titles and abstracts were screened, yielding 13 full-text articles for eligibility assessment. Following full-text review, six studies (total number of participants: 1899) met the inclusion criteria (the remaining articles were excluded for reasons such as irrelevant outcomes or study design). Although this review was planned as a full systematic review, only one outcome could be pooled quantitatively in a meta-analysis due to data heterogeneity (Table 2).

**Table 2:** Included studies MIDAS: Migraine Disability Assessment

Study	Study design	Sample	Type of surgery	Frequency of migraine (before/after surgery)	Severity (pre-/post-surgery scale of 10)	MIDAS score (pre-/post-surgery)
Razeghi et al. (2018) [1]	Prospective controlled	25	Sleeve gastrectomy	11.5 ± 2.3/0.7 ± 0.2	7.0 ± 0.4/1.6 ± 0.5	n/a
Bond et al. (2011) [6]	Prospective observational study	24	Roux-en-Y gastric bypass and gastric band	3.7 ± 3.4/2.2 ± 2.7	6.6 ± 1.8/5 ± 3	22.4 ± 27.3/11.9 ± 28.5
Gunay et al. (2013) [12]	Retrospective cohort study	81	Roux-en-Y gastric bypass	5.7 ± 4.8/na	n/a	n/a
Novack et al. (2011) [13]	Prospective observational	29	Gastric band	6 ± 2.5/1.8 ± 0.9	n/a	21 ± 12.8 /5.9 ± 3.9
Etefagh et al. (2022) [14]	Prospectively collected	60	Roux-en-Y or sleeve gastrectomy	20.9 ± 15.1/8.3 ± 6.5	n/a	64.4 ± 31.9
Nudotor et al. (2021) [8]	A retrospective cohort study	1680	Sleeve gastrectomy and Roux-en-Y gastric bypass	n/a	n/a	n/a

**Figure 2:**
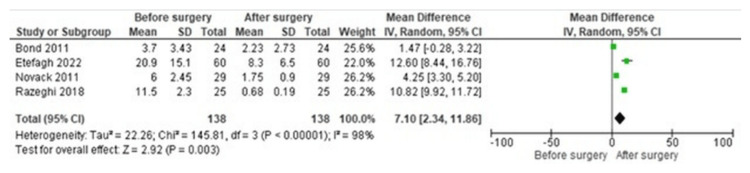
Forest plot demonstrating the reduction in migraine severity following bariatric surgery Each study is labeled with its reference number: Razeghi et al. [1], Bond et al. [6], Novack et al. [13], Etefagh et al. [14]

The forest plots summarizing the meta-analysis demonstrated a consistent pattern of postoperative improvement in migraine outcomes. indicating reductions in migraine frequency with a mean difference of 7.10 and a p-value of 0.03 (Figure 2).

Migraine Frequency

All studies that measured migraine frequency reported a significant postoperative reduction. For example, Razeghi et al. noted a drop from 11.5 ± 2.3 to 0.7 ± 0.2 migraine episodes per month [1]. Similarly, Bond et al. observed reductions from 3.7 ± 3.4 to 2.2 ± 2.7 headaches per month [6].

Migraine Severity

A consistent postoperative decrease in pain intensity was observed across studies. Razeghi et al. reported a reduction from 7.0 ± 0.4 to 1.6 ± 0.5 on a 10-point scale [1]. The forest plot (Figure 2) illustrates these improvements across all included studies.

Migraine-Related Disability

Disability scores (e.g., MIDAS) improved postoperatively in all studies reporting this outcome. Novack et al. [13] demonstrated a decline from 21 ± 12.8 to 5.9 ± 3.9, while Bond et al. [6] showed a drop in moderate-to-severe disability from 50% to 12.5% at six months.

Discussion

Migraine is a common neurological condition that impacts millions of people globally. Its effects are far-reaching, influencing not only the patients but also their families and society as a whole, through both direct and indirect means [8]. The potential mechanisms that might explain the link between migraine headaches and obesity are divided into three main categories: physiological, psychological, and behavioral [6,9]. Various proinflammatory markers that are elevated in obesity, along with adipocytokines such as leptin and adiponectin, have been linked to the experience of migraine pain [9,12]. Despite the increasing evidence of beneficial effects, there remains a lack of research on how bariatric surgeries impact migraine remission. This systematic review evaluates the evidence concerning the impact of bariatric surgery on migraines.

The included studies consistently showed a decrease in the number of migraine days each month, the intensity of pain, and the disability associated with migraines, as assessed by standardized instruments like MIDAS (Migraine Disability Assessment) and HIT-6 (Headache Impact Test-6) [15,16]. Bond et al. observed a decline in average headache days from approximately 11 to about 7 over a 90-day period, along with a reduction in moderate-to-severe MIDAS disability from 50% to 12.5% six months post-surgery [6]. In a similar vein, Novack et al. found a notable decrease in both episodic and chronic migraine occurrences in premenopausal obese women following bariatric surgery, accompanied by enhancements in quality of life scores [13].

Research involving larger sample sizes has validated these results. In a retrospective cohort study of 1,680 patients, Nudotor et al. discovered that 55.4% achieved long-term remission from chronic migraine following surgery, characterized by the absence of migraine medication refills for a minimum of six months [8]. The study also highlighted a slight benefit of Roux-en-Y gastric bypass (RYGB) over sleeve gastrectomy (SG) regarding remission rates. Gunay et al. corroborated these findings in a nine-year retrospective review, revealing that 89% of patients experienced clinical improvement in migraines after RYGB, and more than 70% of those whose migraines developed post-obesity achieved complete resolution [12].

These clinical enhancements might be driven by a mix of neuroendocrine, inflammatory, and behavioral mechanisms. Etefagh et al. presented biochemical evidence by showing a notable decrease in plasma calcitonin gene-related peptide (CGRP) levels after bariatric surgery, a neuropeptide closely linked to migraine pathophysiology [14]. The reduction in CGRP coincided with improvements in both MIDAS scores and patient-reported outcomes, indicating a potential causal relationship. Razeghi et al. further supported this by demonstrating that bariatric surgery resulted in quicker and more significant reductions in the duration and severity of migraine attacks compared to a behavioral weight loss group, even when accounting for the extent of weight loss [1].

Numerous studies have emphasized the anti-inflammatory benefits linked to losing weight. Obesity is connected to increased levels of proinflammatory cytokines, such as IL-6 and TNF-alpha, and adipokines like leptin, which are known to activate the trigeminovascular pathways that play a role in migraines [17]. After surgery, decreases in these biomarkers might lessen systemic inflammation and cortical hyperexcitability, potentially reducing the likelihood of migraines [18]. The metabolic and hormonal changes brought about by bariatric surgeries, including enhanced insulin sensitivity and alterations in gut-brain peptides like GLP-1, may have neuromodulatory effects that further improve migraine management [19].

Behavioral and psychological elements also contribute significantly. Improvements in depression, anxiety, and sleep quality following surgery have been observed in various groups, potentially aiding in migraine relief indirectly [5,13]. Additionally, the decrease in analgesic consumption after surgery, as reported by Novack et al. and Etefagh et al., might help interrupt the cycle of medication overuse headaches, a frequent issue in those with chronic migraines [13,14]. Despite these encouraging findings, several limitations need to be considered. Much of the evidence comes from observational studies, many of which have small sample sizes and lack randomized control groups. Although studies like the one by Razeghi et al. tried to account for confounding factors by including a behavioral weight loss comparison group, the absence of blinding and the possibility of selection bias restrict the ability to draw causal conclusions [1].

Evidence gathered from various studies, which include extensive retrospective analyses and prospective cohort studies, indicates that bariatric surgery plays a beneficial role in managing migraines among obese individuals. For those suffering from persistent migraines alongside obesity, bariatric surgery can provide dual advantages: enhancing metabolic health and alleviating headaches. Notably, the sequence of obesity and migraine onset may hold prognostic significance, as individuals who develop migraines after becoming obese are more likely to see improvement following surgery [5,8,13].

The effect of weight loss and multiple headache parameters was conducted by Hershey et al. [19]. More pronounced reductions in body mass index (BMI) were correlated with more significant decreases in the frequency of headaches at the three- and six-month follow-up periods for children classified as initially overweight or obese, whereas no such relationship was observed in those children who were of normal weight., in addition implication of methods to decrease weight in order to improve weight as A causal association between migraine and obesity may yield significant consequences for healthcare practitioners, patients, and forthcoming public health initiatives, particularly considering the heightened susceptibility to cardiovascular diseases associated with both migraine and obesity [17,18,20,21].

Future investigations should focus on conducting randomized controlled trials that compare bariatric surgery with intensive medical weight loss programs, specifically targeting migraine outcomes. Additionally, longitudinal research is needed to assess the long-term sustainability of migraine remission and to identify factors that predict response, such as initial CGRP levels, gender, and migraine type. Studies examining the mechanistic changes in inflammatory, neuropeptide, and hormonal markers before and after surgery could further elucidate the connections between obesity and migraine.

Limitations

The main limitation of this review is the small number of available studies and the predominance of observational designs, which may introduce selection and reporting bias. Variability in migraine assessment tools, follow-up duration, and types of bariatric procedures limited the quantitative pooling of some outcomes. Although efforts were made to perform a comprehensive search, exclusion of non-English and unpublished data may have led to publication bias. Finally, the inability to perform meta-analysis for all outcomes due to data heterogeneity should be acknowledged. However, the strength of the study lies within the subject matter itself, as contemporary literature predominantly focuses on obesity and cardiometabolic disorders. Nevertheless, it is imperative that scholarly attention be directed towards alternative issues, necessitating innovative and unconventional thought processes.

## Conclusions

In conclusion, bariatric surgery appears to be an effective intervention for reducing the migraine burden in obese patients. Its benefits likely arise from a multifaceted interplay between weight loss, hormonal shifts, inflammation reduction, and behavioral improvements. Given the high comorbidity between migraine and obesity, bariatric surgery should be considered part of a multidisciplinary management strategy for eligible patients.
